# A Dynamic Imaging Simulation Method of Infrared Aero-Optical Effect Based on Continuously Varying Gaussian Superposition Model

**DOI:** 10.3390/s22041616

**Published:** 2022-02-18

**Authors:** Shuyuan Zhang, Xin Chen, Yingqing Zu, Peng Rao

**Affiliations:** 1 Key Laboratory of Intelligent Infrared Perception, Chinese Academy of Sciences, Shanghai 200083, China; zhangshuyuan@mail.sitp.ac.cn (S.Z.); chenxin@mail.sitp.ac.cn (X.C.); 2 Shanghai Institute of Technical Physics, Chinese Academy of Sciences, Shanghai 200083, China; 3 University of Chinese Academy of Sciences, Beijing 100049, China; 4 Department of Aeronautics and Astronautics, Fudan University, Shanghai 200433, China; yzu@fudan.edu.cn

**Keywords:** aero-optical effect, infrared imaging degradation, point spread function

## Abstract

Aero-optical effect correction has become a crucial issue in airborne infrared imaging. However, it is impractical to test the correction algorithm using flight tests and numerical simulation because of its high cost. This study proposes a dynamic imaging simulation method for the infrared aero-optical effect based on a continuously varying Gaussian superposition model. The influence of infrared image degradation under different high-speed aerodynamic flow fields was investigated in detail. A continuously varying Gaussian superposition model was established for flight speed, altitude, and attitude. A dynamic infrared scene simulation model was constructed. Experimental results show that the proposed method can realistically simulate actual aero-optical effects of any flight case. Moreover, it can simulate continuous frames of aerodynamically degraded infrared images. The method uses a simpler model than numerical simulation and provides more data for multitype tasks.

## 1. Introduction

Airborne infrared (IR) optical systems play an important role in airborne remote sensing. Aerodynamic optical distortion has become a serious concern in imaging systems with the development of aerospace technology [1,2,3,4,5,6,7,8]. Large density gradients of turbulent compressible flow around the optical window are the direct reasons for aero-optical distortions. The flow field outside the optical window may include complex flow structures, such as the free shear layer, turbulent boundary layer, and shock wave. The distribution of its density gradient is unstable and changes constantly. According to the Gladstone–Dale relationship, the index of refraction in the air is proportional to its density. Severe wavefront distortion arises in the beam through the aerodynamic flow field owing to variations in the index of refraction, leading to blurring, light deflection, and jitter. These are referred to as aero-optical effects; they critically affect the imaging quality of airborne optical systems [9]. Digital image correction technology can improve the quality and accuracy of airborne infrared remote sensing tasks. Research on traditional image processing algorithms and deep learning algorithms all rely on an abundance of data. Therefore, it is necessary to study imaging changes caused by high-speed flight conditions to obtain degraded image data.

The imaging distortion caused by the aero-optical effect has attracted significant attention for many decades. Recent developments have been summarized in Refs. [10,11,12]. Liu et al. [13] investigated the influence of a non-uniform aerodynamic flow field and an aerodynamically heated optical window on the imaging quality. Xu et al. [14] studied the imaging deviation in two flying conditions by proposing a backward ray-tracing scheme. The numerical calculation of the flow field and optical transmission is a commonly used method to estimate the transmission effect. Such a method uses computational fluid dynamics software with slow computing speed; hence, it can only be used for limited flight cases. The Airborne Aero-Optics Laboratory (AAOL) provides an airborne platform that can be used for subsonic and transonic in-flight testing. Kalensky et al. [15] recorded visible-light distortion images in realistic flight environments based on AAOL with the Mach number up to 0.7, which cannot simulate the shock layer in a high-speed flow field. Additionally, IR imaging requires specialized cameras and optics. Therefore, the use of flight tests cannot obtain a large amount of serial IR aerodynamic degradation image data at present, considering the expensive research cost.

Considering that an IR imaging system is influenced by atmospheric turbulence, Frieden [16,17] described the point spread function as a stochastic superposition model that follows the true physics of turbulence. Frieden used fixed parameters for the model. Banish et al. [18] evaluated the point spread function for a short-wavelength strong turbulent system; they found that the shape of the turbulence point spread function is a Gaussian distribution. Xiao et al. [19] introduced the shock wave density formula for supersonic flight to build an expression to describe the relationship between the size of the point spread function and prior flight parameters. Xiao et al. [20] empirically determined the range of values of the number, size, and position of turbulence units through numerous wind tunnel experiments. Zhang et al. [21] set the turbulence unit parameters of the stochastic superposition model to change randomly within a certain range over time. However, the intensity-controlled parameters of the model must be set separately based on empirical values. Therefore, the model is only suitable for describing the law of image quality degradation and cannot perform real-time simulation based on flight status. Hence, it is necessary to further promote research on real-time simulation of the IR aero-optics effect to explore aero-optics and its real-time correction efficiently and at a low cost.

**Figure 1:**
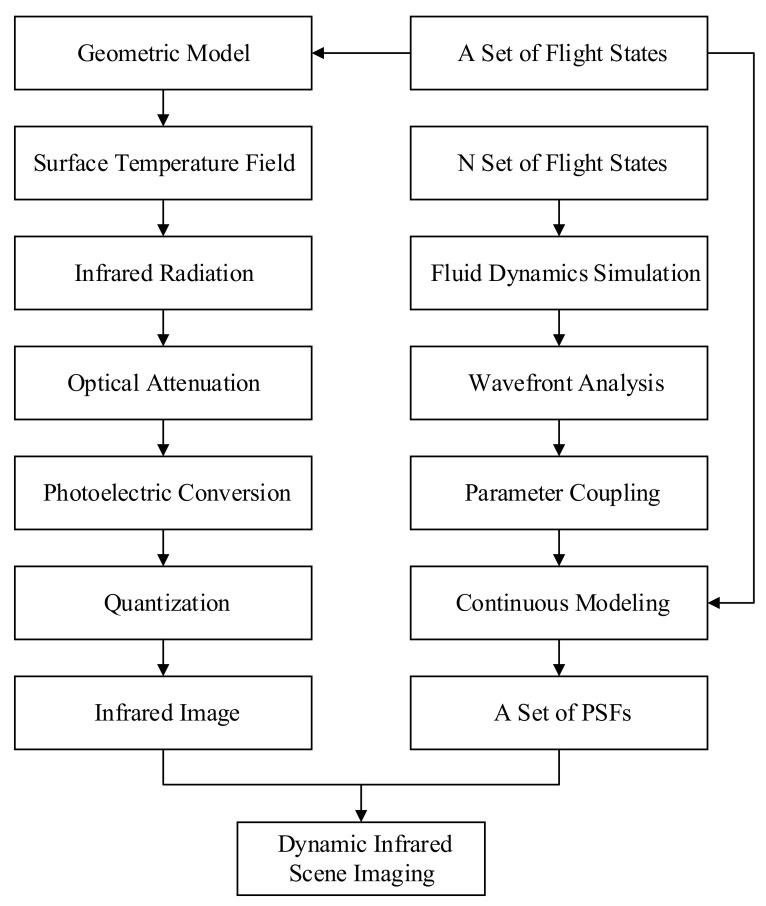
Flowchart of the proposed method.

This paper presents a study of the influence of aerodynamic optical transmission based on fluid dynamics simulation and wavefront analysis. The mapping relationship between the aerodynamic transmission intensity parameters and flight parameters was obtained through regression analysis. Then, a Gaussian mixture superposition continuous degradation model was established. Additionally, we simulated the imaging process of a real IR detection scene, combined with the continuous degradation model, to develop the full-link imaging simulation of IR aerodynamic degradation. The entire framework of the proposed method is shown in Figure 1. Modulation transfer function (MTF), point spread function (PSF), and the values of image quality evaluation index—peak signal-to-noise ratio (PSNR) and average gradient (GMG)—were used to evaluate the aero-optical transmission effect of the continuously varying Gaussian superposition degradation model. 

The remainder of this paper is organized as follows. Section 2 introduces the modeling methods of IR scene imaging simulation. Section 3 builds a continuous degradation model of the aero-optical effects coupled with flight parameters. Section 4 presents the results and analysis. Finally, Section 5 concludes the paper.

## 2. IR Scene Simulation

The IR detection scene simulation simulates the process of transforming the IR radiation of the target and the background into gray values through the detector and displays it as an image.

We used 3D MAX to establish a three-dimensional geometric model of the target and background. The surface temperature of each part of the scene was set. The position of the target on the image was obtained according to the spatial position of the aircraft. 

Spectral radiance can be obtained according to Planck’s law. The radiance corresponding to the response band of the detector was calculated using the following integral:

(1) $$L\left(\lambda ,T\right)=\frac{1}{\pi }\int_{\lambda_{1}}^{\lambda_{2}}\epsilon \frac{c_{1}}{{\pi \lambda }^{5}}\frac{1}{e^{c_{2}/\lambda T}\;-\;1}d\lambda ,$$

where T denotes the target surface temperature, ϵ represents the emissivity of the target surface material, c_1_ is the first radiation constant ($3{.7415\;\times \;10}^{4}{\;W\cdot \mathrm{cm}}^{-2}{\cdot \mu m}^{4}$), and c_2_ is the second radiation constant ($1{.43879\;\times \;10}^{4}\;\mu m\cdot K$).

The output voltage can be expressed as follows [22]:

(2) $${V=P\cdot A}_{d}\cdot J,$$

where P represents the sensitivity of the detector, $A_{d}$ denotes the pixel area of the detector, and  J is the radiance of the target.

(3) $$J=\frac{L\cdot \mathrm{opttran}\cdot {\left(D/2\right)}^{2}}{f^{2}},$$

where L represents the radiance of the target, opttran denotes the optical transmittance of the optical system, D is the aperture of the optical system, and f is the focal length of the optical system.

The voltage value was quantified into the gray value of the image using the following formula [22]:

(4) $${G=G}_{\min}+\frac{\left(V_{H}{\;-\;V}_{L}\right)\left(G_{\max}{\;-\;G}_{\min}\right)}{V_{H}{\;-\;V}_{L}},$$

where $(G_{\min},{\;G}_{\max}$) represents the grayscale quantization range; $V_{L}\;$ and $V_{H}$ denote the minimum and maximum output voltages, respectively.

## 3. Aero-Optically Continuous Degradation Model

### 3.1. Aero-Optical Numerical Simulation

The high-speed flow field simulation procedure based on physical characteristics generally starts by analyzing the flow field characteristics, and then the impact on imaging is analyzed. It includes two main parts: flow field simulation and optical simulation. The methods of estimating the transmission effect of the turbulent flow field include Reynolds average Navier–Stokes (RANS) [23], direct numerical simulation (DNS) [24], and large eddy simulation (LES) [25]. Optical simulation primarily uses optical methods, including geometric optics, physical optics, and statistical optics, to calculate and analyze the distortion of light after it passes through the flow field. 

**Figure 2:**
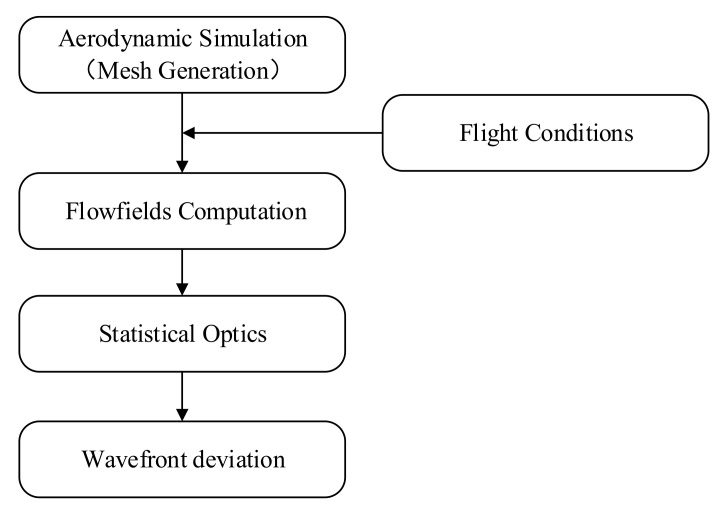
Flowchart of the aero-optical transmission analysis.

In this section, the computational fluid dynamics (CFD) technology was used to simulate the near-field non-uniform flow field under different conditions. Then, the density distribution on the IR optical path was obtained. Thereafter, the refractive index field based on the linear relationship between the density and refractive index was measured. Finally, the wavefront deviation of the optical system was calculated using statistical optics. The flowchart of aero-optical transmission analysis is shown in Figure 2.

**Figure 3:**
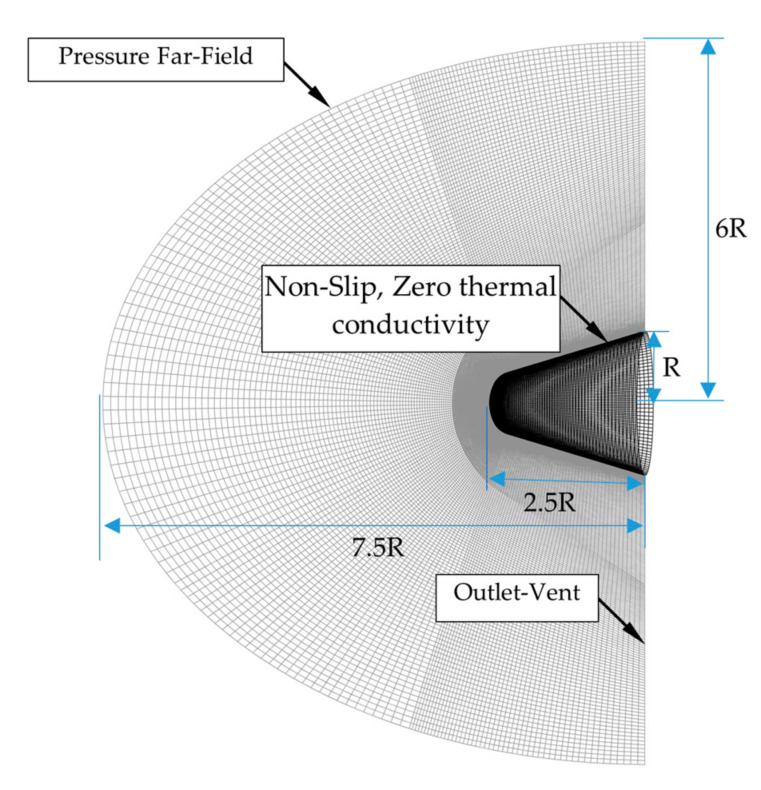
Mesh and boundary conditions of computational domain.

We analyzed the flow around the head of a spherical window. It was basically a cone with a hemisphere at the top as shown in Figure 3. The optical window was set above the hemisphere; the pupil was located at the center of the window. The structure behind the aircraft head was not considered because only the external flow field near the optical window was needed for the flow to be analyzed. The computational domain is axisymmetric. The mesh at the middle cross-section of flow field and the surface of the aircraft head are shown in Figure 3. As shown in the figure, structured multi-block grids were generated in the computational domain. C-type grids were applied to increase the orthogonality of the mesh. The thickness of the first layer of elements adjacent to the surface of the aircraft head was set as 0.001 mm and the grid expansion ratio was set as 1.2 so as to obtain the accurate velocity boundary layers and, meanwhile, to ensure wall y+ < 1. The mesh in the vicinity of the aircraft was further refined to capture shock wave with high resolution. Based on the grid-independent test, the mesh with 1.76 million hexahedral elements was adopted for all of the following simulations. The boundary conditions used for the simulation were referred from the work of Sundarraj et al. [26]. As shown in Figure 3, a pressure far-field boundary condition was used to model a free-stream compressible flow at infinity, with free-stream Mach number and static pressure and static temperature specified. Here, the static pressure and static temperature conditions were given on the basis of the ambient temperature and pressure, which were derived from the data of U.S. standard atmosphere [27] for the specified flight altitudes. In addition, the Mach number was given on the basis of the flight speed and attack angle. The specific parameters are seen in Appendix A. The surface of the aircraft was set to be non-slip and zero thermal conductivity. The other boundary was set to be outlet-vent with ambient static pressure and temperature. 

The aircraft flies in the atmosphere at high speed and interacts with the incoming flow. Kinetic energy is transformed into a high thermal environment and a complex flow field owing to friction and compression effects. The commercial CFD solver Fluent R2021 was used to simulate the aerodynamic thermal environment, considering the fluid as a continuous thermal medium and output the distribution of key physical quantities, such as the temperature, density and the turbulence parameters. The solver was set to be density-based, implicit and steady. The finite volume method was employed to discretize the RANS equations; the realizable k–ε turbulence model [28] was employed to close the equations. The standard wall functions were applied to model the flow field in the near-wall region. The AUSM+ flux scheme [29] was used for shock-capturing. Second-order upwind scheme was adopted to discretize the mass, momentum and energy equations. In addition, the QUICK scheme was used to discretize the transport equations of turbulence kinetic energy k and the turbulent energy dissipation rate ε.

**Figure 4:**
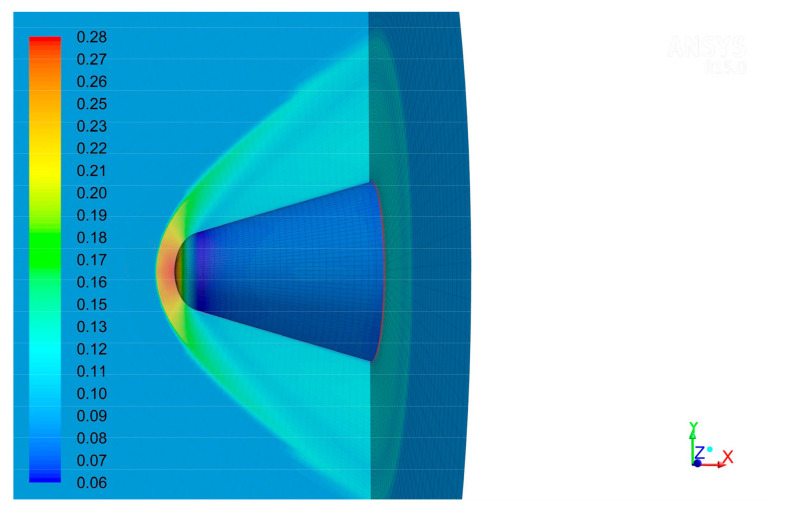
Density contours (kg/m^3^) of the flow field at 20 km altitude, 2 Mach number.

Figure 4 shows the density contour of the flow field under the flight condition: flight height H=20 km, flight velocity Ma=2, and angle of attack α=0°. The off-body shock wave appeared ahead of the optical window. The density of flow field distributed axisymmetrically. Our finding is qualitatively in line with that of previous studies [26].

The refractive index and density of the fluid obey the Gladstone–Dale relation, transforming the density distribution into a refractive index distribution.

(5) $$n\left(x,y,z\right){=1+K}_{\mathrm{GD}}\rho \left(x,y,z\right),$$

where n  and ρ  denote the index of refraction and density, respectively. The Gladstone–Dale coefficient $K_{GD}$ is a constant and depends weakly on the wavelength λ (μm) [30]. It can be approximated as follows:

(6) $$K_{\mathrm{GD}\;}\approx \;2{.24\;\times \;10}^{-4}\left(1+\frac{7{.52\;\times \;10}^{-3}}{\lambda^{2}}\right)\left(m^{3}/\mathrm{kg}\right),$$

The optical transmission was simulated using the statistical optical method [31]. The mean square error of refractive index fluctuation can be expressed by the following formula:

(7) $$\langle {\left(n_{\prime }\right)}^{2}\rangle =C\cdot \frac{k^{3}}{\varepsilon^{2}}\cdot {\left(\nabla n\right)}^{2},$$

where k is the turbulent kinetic energy, ε is the turbulent energy dissipation rate , C is an empirical constant with values of 0.3528 [32,33,34].

The correlation scale of turbulence pulsation can be approximated as follows [34]:

(8) $$l_{c}=\frac{k^{\frac{3}{2}}}{\varepsilon }.$$

The root-mean-square (RMS) phase error is used to measure wavefront distortion. It can be approximated as follows [35]:

(9) $$\sigma =\sqrt{2k^{2}\int_{0}^{L}\langle {\left(n_{\prime }\right)}^{2}\rangle l_{c}\mathrm{dz}}.$$

where z is the axis coordinate direction of fluent, and l is the optical path distance through the fluid to the window.

Strehl ratio (SR) was chosen to evaluate wavefront distortion:

(10) $$\mathrm{SR}=\exp(-{\left(<\sigma >\right)}^{2}).$$

where <σ> is the mean value of the phase error.

### 3.2. Continuously Varying Degradation Model

The continuous model is based on Frieden’s superposition model, which also abides by the turbulence Kolmogorov law [20]. It can simulate the actual impact of aero-optical effects on imaging, as follows:

(11) $$s\left(r\right)=\sum_{m=1}^{M}\omega_{m}{h(r\;-\;r}_{m}),$$

where h(r) represents the turbulence unit disturbance function, $\omega_{m}$ represents the weight of each turbulence unit, M represents the number of turbulence units, and $r_{m}$ denotes the position change. 

The turbulence unit superimposes the intensity through random weights and realizes a random distribution in space. The disturbance function of the turbulence unit is Gaussian [18].

(12) $$\mathrm{PSF}=\exp\left[\;-\;\frac{\left({\left({x\;-\;x}_{m}\right)}^{2}+{\left({y\;-\;y}_{m}\right)}^{2}\right)}{{2\sigma }_{m}{}^{2}}\right],$$

where ($x_{m}$,${\;y}_{m}$) represents the displacement vector of the disturbance function, and $\sigma_{m}$ is the ambiguity factor that measures the wavefront error. The size of $\sigma_{m}$ is the comprehensive scale of each small turbulence, determining the intensity of the disturbance function.

The number of turbulence units varies randomly with time. We set random weights for the number of small turbulences to ensure randomness. According to theoretical analysis, the amount of small turbulence is directly proportional to the magnitude of the speed and inversely proportional to the flying height [20]. The angle of attack, which changes to a small extent, exhibits little effect on the number of turbulence units. Therefore, the number of turbulence units is obtained as follows:

(13) Number=μ·Ma/H.

where μ is the empirical random weight with a value range of 5 to 10 [20]. 

The coordinates $\left(x_{\max},y_{\max}\right)$ corresponding to the maximum value can be obtained by measuring the maximum value of the PSF distribution. Based on this, the overall image shift caused by the aerodynamic transmission effect can be estimated, i.e., the image deviation in the x-direction and y-direction is $x_{\max}$ and $y_{\max}$, respectively. Each turbulence unit was randomly distributed in the action area. Furthermore, the displacement vector should be a random number in the range of (−R/2, R/2). The center coordinate of the main peak was considered as the starting point because the small turbulence was distributed around the main peak. The small turbulence coordinate is expressed as $\left(R_{1},\;\theta \right)$ in the polar coordinate system [21].

(14) $$x_{m}=\frac{R}{2.0}+R1\cos\;\left(\theta \right)+R2\mathrm{Gause}\left(0,1\right),\;y_{m}=\frac{R}{2.0}+R1\sin\;\left(\theta \right)+R2\mathrm{Gause}\left(0,1\right),$$

where R1  represents the distance from the center of the turbulence unit to the center of the main peak. R2 denotes a probability parameter that controls the degree of discrete distribution of small turbulence. The greater the R2, the greater is the jitter degree of the small turbulence coordinate.

The overall model is as follows:

(15) $$P\mathrm{SF}=\sum_{m-1}^{M}\omega_{m}\exp\left[-\frac{\left({\left({x\;-\;x}_{m}\right)}^{2}+{\left({y\;-\;y}_{m}\right)}^{2}\right)}{{2\sigma }_{m}{}^{2}}\right].$$

The weight $\omega_{m}$ was set as a normally distributed random number, reflecting the random distribution characteristics of the turbulence unit. Moreover, the size of the weight $\omega_{m}\;$ is inversely proportional to the distance between the turbulence unit and the main peak because the turbulence unit distributed closer to the turbulence center has a larger influence component.

### 3.3. Analysis of the Intensity Factor

The flight speed was increased from 1 Ma to 4 Ma, and the flight altitude was reduced from 10 km to 1 km. The attitude was observed within a small range (0–8°). See the appendix for specific parameter values. We can obtain the RMS phase error (σ) under different flight conditions based on the flow field simulation and wavefront calculation presented in Section 3.1.

We analyzed the mean value of the  σ that varies with flight speed, altitude, and state to obtain a continuous degraded function coupled with flight parameters. The size of the samples was small. To avoid overfitting, we used 10-fold cross-validation to divide the training set and the test set, which could make full use of the existing samples. The samples were divided into ten parts. One of the ten parts was used as the test set each time. The process was repeated until all the data had been tested. Additionally, we augmented the test set by adding noise. The signal-to-noise ratios of noise were 20, 30 and 40, respectively. The test set was expanded to 100.

**Figure 5:**
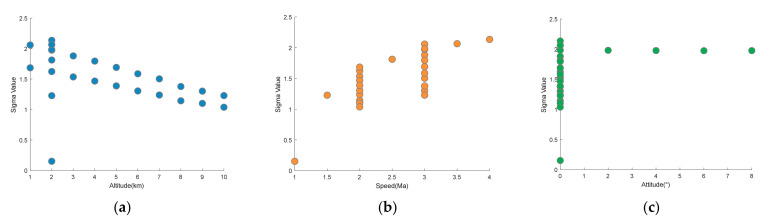
Scatter diagram of the value of σ versus flight altitude, speed, and attitude: (**a**) σ values varying with altitude; (**b**) σ values varying with speed; (**c**) σ values varying with attitude.

First, sample distribution should be observed to guide analytical decisions. Scatter diagrams are shown in Figure 5. As seen in the Figure 5, the observation points of each parameter exhibit a clear change trend within the current parameter range. Then, we calculated the correlation coefficient between σ and the flight parameters to obtain the correlation between variables. Table 1 shows the results of the significance tests. We observed that the flight altitude showed a negative correlation, the flight speed showed a strong positive correlation, and the flight attitude changed positively in the current range without the strong correlation. These results were consistent with the preliminary theoretical analysis; therefore, we used the value of σ as blurred factor ($\sigma_{m}$) of the point spread function.

According to the distribution of samples, we calculated statistical measure results of the linear model, exponential regression model, power regression model, and power regression model without outliers. An outlier is a data point with an extreme value or one that differs significantly with other cases [36]. Outliers carry a negative impact on the regression results; therefore, checking the presence of outliers is necessary. The outliers were judged and screened out by calculating the 95% confidence interval of the residuals.

Mean squared error (MSE), mean absolute error (MAE), and coefficient of determination (R squared or $R^{2}$) are commonly used statistical measures for model performance evaluation. $R^{2}$ is a ratio about the model error with baseline error, which ranges from 0 to 1. A value close to 1 indicates the reliable predictive performance of the model. $R^{2}$ can be expressed by the following formula [37]:

(16) $$R^{2}=-\frac{\sum_{i=1}^{m}{\left(X_{i\;}{-\;Y}_{i}\right)}^{2}}{\sum_{i=1}^{m}{\left(\bar{Y\;}{-\;Y}_{i}\right)}^{2}},\;$$

where X and Y are predicted values and observed values, respectively, and  Y ¯ is the mean value of the Y. 

Table 2 shows the comparison of the statistical measure results of different regress models. To ensure accuracy, we performed 10-fold cross-validation with 100 times and then averaged the results. The results showed that the MSE and MAE of the power regression after excluding the abnormal points were smaller than the results of other models, and that the $R^{2}$ was the highest among the results of other regress methods. It illustrated that the power regression model with outliers eliminated can produce reliable prediction results.

## 4. Results and Discussion

### 4.1. Wavefront Distortion Analysis of the Numerical Simulations

Flight cases mainly contained the following parameters within the value range: flight altitude, H=1–10 km; flight speed, Ma=1–4; flight attitude, A=0–8°. The attitude of the flight is the angle of attack, which represents the angle between the flight direction and the axis of the vehicle. The following were the optical and detector parameters: wavelength is 4 μm; focal length is 88 mm.

Table 3 shows the SR results under twenty no-overlap states. From Table 3, the relationship between the aero-optical effects and the flight conditions was revealed: (1) The SR value decreased as the Mach number increased. Because the density gradient of flow fields increased along with the Mach number, it led to the influence of increased aero-optical effects. (2) With the increase of flying height, the non-uniformity of flow field density decreases significantly. Thus, the SR value decreases with the increase of height. The influence of the aero-optical effect will increase with the increase of flying height. (3) As the attitude angle increases, the SR value becomes smaller, which means that the aero-optical effect will become more serious as the angle increases. However, the effect of attitude is much smaller than that of altitude and speed.

### 4.2. Influence of Speed and Altitude on Aerodynamic Transmission Effect

**Figure 6:**
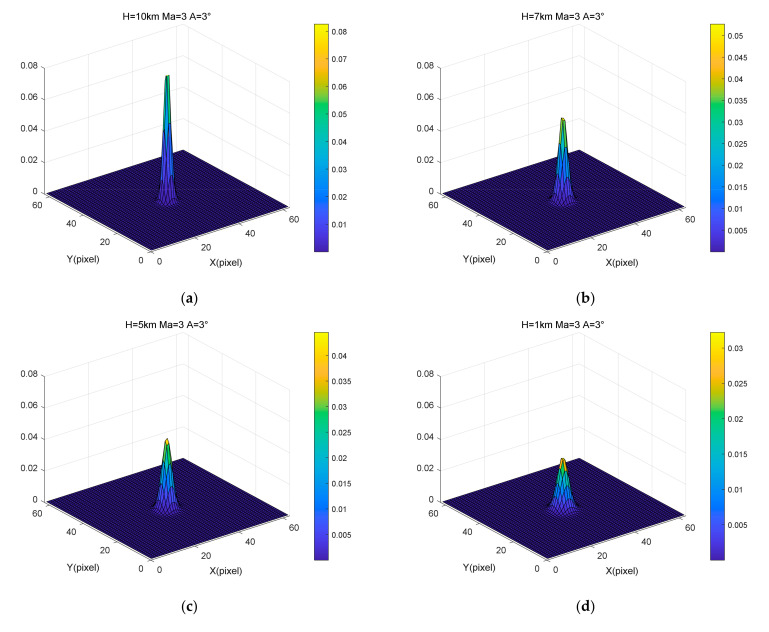
Variations in PSF results with different heights: (**a**) Mach number = 3, Angle of attack = 3°, Height = 10 km, (**b**) Mach number = 3, Angle of attack = 3°, Height = 7 km, (**c**) Mach number = 3, Angle of attack = 3°, Height = 5 km, (**d**) Mach number = 3, Angle of attack = 3°, Height = 1 km.

**Figure 7:**
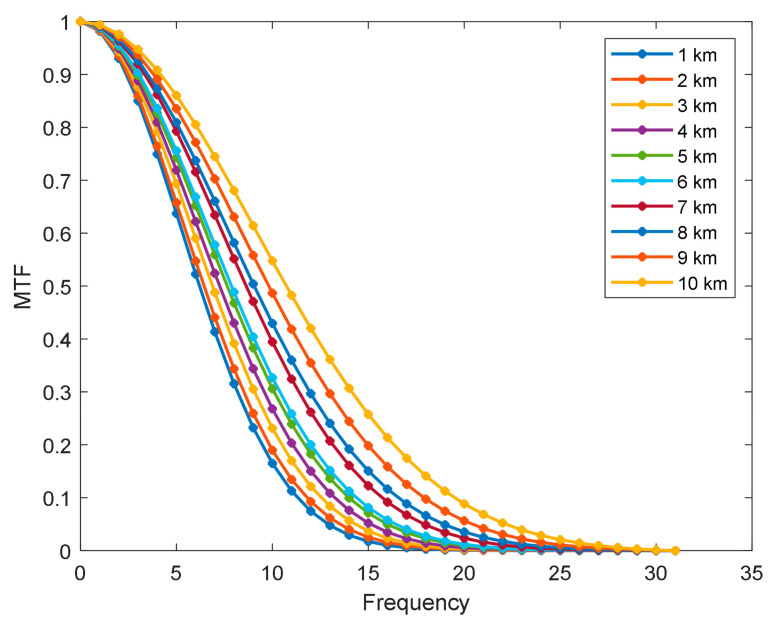
Change in MTF results with different heights.

The PSF results generated by the continuously varying Gaussian superposition model at different heights are shown in Figure 6. The heights were 10 km, 7 km, 5 km, and 1 km. Additionally, the speed and angle of attack were 3 Ma and 3°, respectively. The Fourier transform of the PSF results in the optical transfer function, the modulus of which is the MTF. The MTF results for different heights are shown in Figure 7. The aero-optical effect does not change significantly within a small angle of attack; the optical window is axisymmetric; the MTF values in the meridian and sagittal directions primarily coincide. All MTF curves were obtained in the radial direction to make the results more intuitive. According to these figures, the amplitude of the PSF decreases as the flight altitude decreases, increasing the energy range; the MTF decreases faster as the flight altitude decreases. These results show that the intensity of the aero-optical effects gradually increased with decreasing height decreases, causing the image quality to degrade gradually.

**Figure 8:**
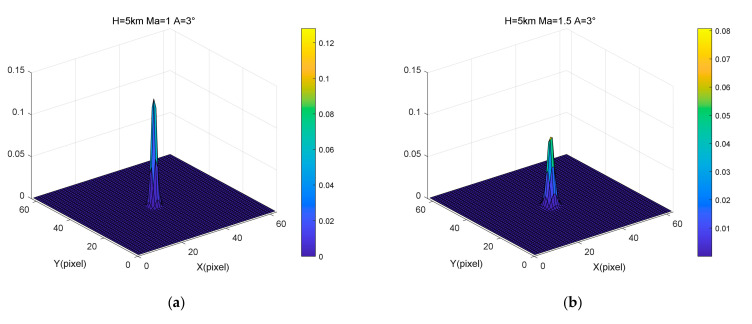

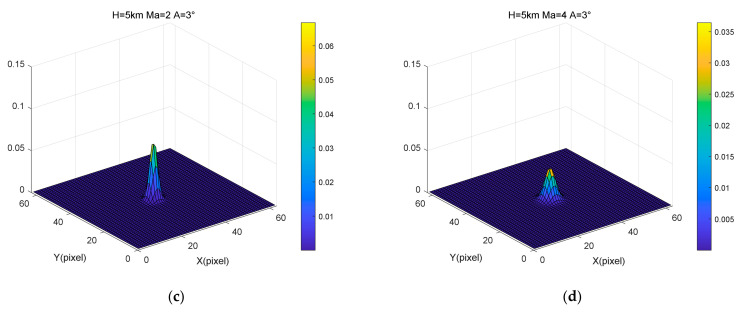
Variations in PSF results with different velocities: (**a**) Mach number = 1, Angle of attack = 3°, Height = 5 km, (**b**) Mach number = 1.5, Angle of attack = 3°, Height = 5 km, (**c**) Mach number = 2, Angle of attack = 3°, Height = 5 km, (**d**) Mach number = 4, Angle of attack = 3°, Height = 5 km.

**Figure 9:**
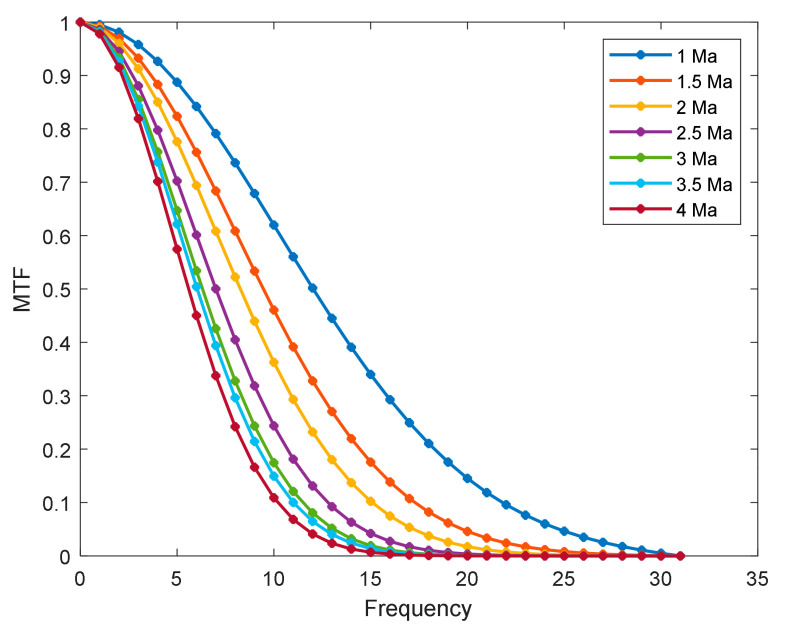
Variations in MTF results with different velocities.

Figure 8 illustrates the PSF results generated by the continuously varying degradation model under four Mach numbers: 1 Ma, 1.5 Ma, 2 Ma, and 4 Ma. Furthermore, the height and angle of attack were 5 km and 3°, respectively. Figure 9 shows the MTF results for different Mach numbers. Figure 8 and Figure 9 show that, as the Mach number became larger, the amplitude of the corresponding PSF became smaller, and the energy range became larger, with the MTF decreasing faster. The faster the flight flies, the more aerodynamic transmission affects image quality.

**Figure 10:**
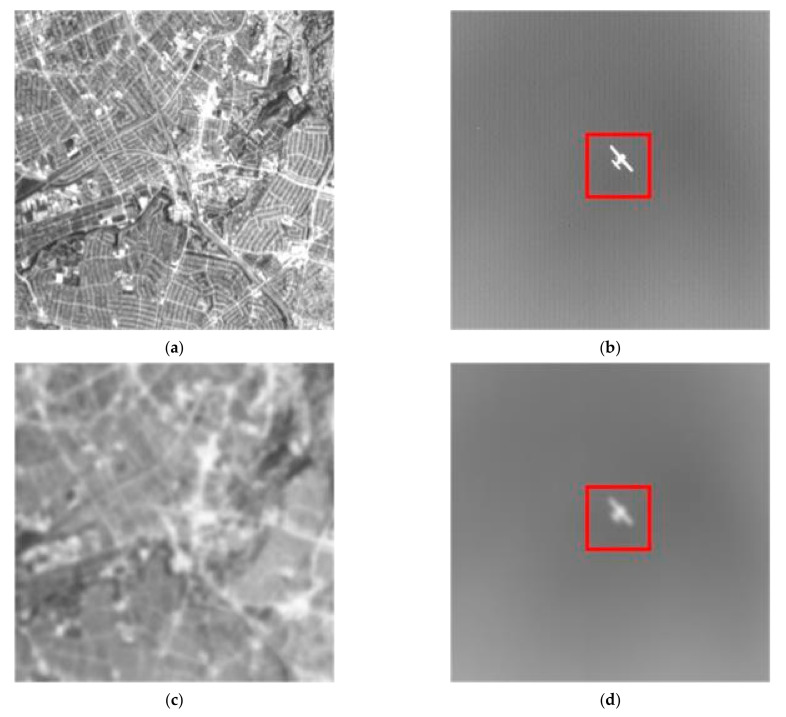
Original image (**a**) with the complex background; original image (**b**) with simpler background and target; distorted imaging result (**c**,**d**).

We convolved the continuously degraded model with the original images to obtain the simulated degraded images. Figure 10a,b [38] show the captured original mid-wave IR images. Figure 10a is a complex background, and Figure 10b is a simple background. Figure 10c,d show the imaging results with the addition of aero-optical effects. We used the PSNR and GMG to evaluate the quality of the image. We only measured the image quality of the red box in Figure 10b to obtain clearer results. Table 4 shows the changes in the quality evaluation index of Figure 10a,b with the flight altitude and speed. The results show that the PSNR and GMG of the image gradually decreased with increasing the flight speed and decreasing flight altitude. It shows that the image became blurred compared to the original image, weakening the edge details and texture.

### 4.3. Simulation of Dynamic Aerodynamically Degraded Infrared Images

Under the assumption of a spatially invariant blur, the aerodynamic imaging degradation model can be expressed as follows:

$$g\left(x,y\right)\;=\;k\left(x,y\right)⊗f\left(x,y\right)\;+\;n\left(x,y\right),$$

where f denotes the IR simulation image, k represents the degrading model caused by the aero-optical effect, n is the additive noise, g denotes the degraded image, and ⊗ represents the convolution operation.

**Figure 11:**
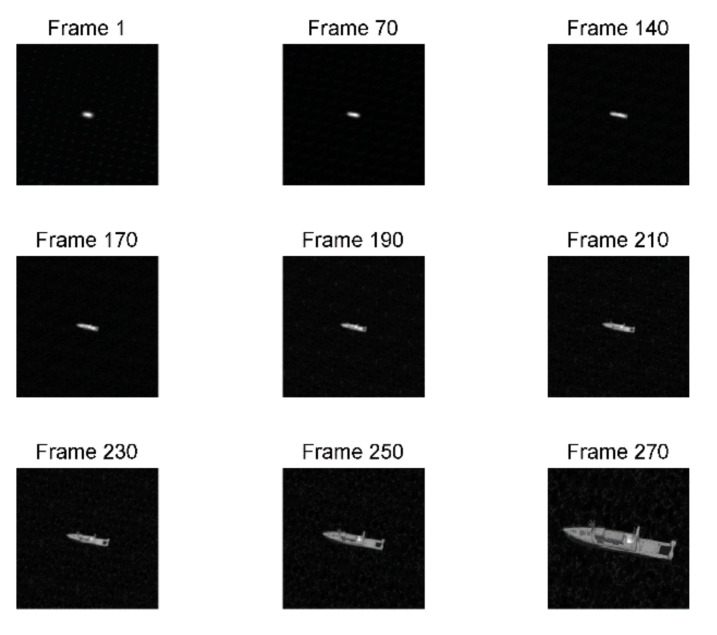
A sequence of IR aerodynamic degradation images.

The image degradation model affected by aero-optical effects includes two parts: the aerodynamic flow field degradation model and the aerodynamic thermal radiation degradation model. For simplicity, we only considered the impact of the aerodynamic flow field transmission effect. We obtained the aerodynamically degraded IR images sequence in different scenes and flight states based on the IR detection scene simulation model in Section 2 and the continuous degradation model of the aero-optical effect in Section 3. Our method can simulate continuous multiple frames of IR aerodynamic degradation images. Figure 11 shows nine frames captured from the sequence of the IR aerodynamic degradation images.

## 5. Conclusions

The aerodynamic turbulence causes serious degradation of IR imaging. Therefore, image correction is one of the most important research topics in aero-optical effects. However, it is difficult to provide a large amount of distortion images for the research of real-time correction algorithms owing to the cost limitation of current methods. In this paper, the wavefront distortion of different high-speed flow fields through numerical simulation was obtained firstly. Second, the flow field intensity factors under different flight parameters were obtained by combining the trajectory and attitude of the aircraft. Then, an aero-optically continuous degradation model was established. Furthermore, we built a full-link IR imaging degradation simulation model for the first time, which can generate sequential IR aerodynamic degradation images.

The influence of speed and altitude on the aerodynamic transmission effect was analyzed from the following three aspects: PSFs, MTFs, and image quality evaluation indicators. Analysis results show that the continuous degradation model can reflect the changes of aero-optical effects with flight altitude and speed: the faster the flight flies, the lower the flight height, the more aerodynamic transmission affects image quality. Our conclusions are consistent with that of the theoretical analysis and numerical simulation. Therefore, the dynamic imaging simulation can provide a priori information to guide the research on real-time correction algorithms, provide a large amount of data for deep learning, and can also be used as a ground verification platform for real-time correction algorithms.

## Figures and Tables

**Table 1:** Results of the significance test.

Flight Parameters	Speed	Altitude
R-value	0.7639	−0.5226
*p*-value	7.525 × 10^−7^	0.004

**Table 2:** Statistical measure results of different regress models.

Flight Parameters	MSE	MAE	R^2^
linear regression model	0.0631	0.1531	0.83
exponential regression	0.1118	0.1812	0.74
power regression model	0.0493	0.1377	0.89
power regression model without outliers	0.0406	0.0753	0.93

**Table 3:** SR under different flight cases.

Mach number	1	1.5	2	2.5	3	3.5	4
Altitude (km)	2	2	2	2	2	2	2
Attitude (°)	0	0	0	0	0	0	0
SR	0.9774	0.2219	0.0718	0.0375	0.0197	0.0197	0.0105
Mach number	3	3	3	3	3	3	3
Altitude (km)	1	3	4	5	6	7	8
Attitude (°)	0	0	0	0	0	0	0
SR	0.0145	0.0293	0.0399	0.0574	0.0809	0.1045	0.1502
Mach number	3	3	3	3	3	3	
Altitude (km)	9	10	2	2	2	2	
Attitude (°)	0	0	2	4	6	8	
SR	0.1839	0.2213	0.0198	0.0201	0.0203	0.0204	

**Table 4:** PSNR and GMG of distorted images with the changes in flight speed and altitude.

Image	Radio	H1Ma3	H5Ma1	H5Ma2	H5Ma3	H7Ma3	H9Ma3
Figure 10a	PSNR	21.04	25.10	22.93	21.60	21.92	22.32
	GMG	3.95	10.18	6.48	4.62	5.01	5.52
Figure 10b	PSNR	24.26	28.80	26.42	24.92	25.28	25.76
	GMG	1.70	3.42	2.39	1.87	1.98	2.11

## Appendix A

**Table A1:** The specific parameter values.

Mach Number	Altitude (km)	Attack Angle (°)
1	2	0
1.5	2	0
2	1	0
2	2	0
2	3	0
2	4	0
2	5	0
2	6	0
2	7	0
2	8	0
2	9	0
2	10	0
2.5	2	0
3	1	0
3	2	0
3	3	0
3	4	0
3	5	0
3	6	0
3	7	0
3	8	0
3	9	0
3	10	0
3	2	2
3	2	4
3	2	6
3	2	8
3.5	2	0
4	2	0

## References

[1] Zhao X., Yi S., Ding H. (2021). Aero-optical testing of a Mach 3 cooling film. Optik.

[2] Guo G., Luo Q., Gong J. (2021). Evaluation on aero-optical transmission effects caused by a vortex in the supersonic mixing layer. Opt. Commun..

[3] Guo G., Zhu L., Bian Y. (2020). Numerical analysis on aero-optical wavefront distortion induced by vortices from the viewpoint of fluid density. Opt. Commun..

[4] Ding H., Yi S., Ouyang T., Shi Y., He L. (2020). Influence of turbulence structure with different scale on aero-optics induced by supersonic turbulent boundary layer. Optik.

[5] Niu Q., Gao P., Yuan Z., He Z., Dong S. (2019). Numerical analysis of thermal radiation noise of shock layer over an infrared optical dome at near-ground altitudes. Infrared Phys. Technol..

[6] Sun C., Wang Z., Cheng X., Xia X. (2018). Simulation and evaluation of stray radiation from optical window effects on infrared detection system. Infrared Phys. Technol..

[7] Emelyanov V., Teterina I., Volkov K., Yakovchuk M. (2018). Aero-optical effects in free and wall-bounded turbulent compressible flows. Acta Astronaut..

[8] Liu L., Meng W., Li Y., Zuo Z., Dai X. (2014). Analysis and modeling of aerothermal radiation based on experimental data. Infrared Phys. Technol..

[9] Li G. (2006). Aero-Optics.

[10] Sun X., Liu W. (2020). Research progress of aero-optical effect. Adv. Mech..

[11] Jumper E.J., Fitzgerald E.J. (2001). Recent advances in aero-optics. J. Prog. Aerosp. Sci..

[12] Jumper E.J., Gordeyev S. (2017). Physics and Measurement of Aero-Optical Effects: Past and Present. Annu. Rev. Fluid Mech..

[13] Liu L., Meng W., Li Y., Dai X., Zuo Z. (2015). Influence of aero-optical transmission on infrared imaging optical system in the supersonic flight. Infrared Phys. Technol..

[14] Xu L., Cai Y. (2012). Imaging deviation through non-uniform flow fields around high-speed flying vehicles. Optik.

[15] Kalensky M., Wells J., Gordeyev S. (2020). Image degradation due to different in-flight aero-optical environments. Opt. Eng..

[16] Frieden B.R. (1994). Turbulent image reconstruction using object power spectrum information. Opt. Commun..

[17] BFrieden B.R., Oh C. (1993). Turbulent image reconstruction from a superposition model. Opt. Commun..

[18] Banish M., Clark R., Kathman A. A validated code to predict the performance of onboard broadband optical seekers through a turbulent transonic flow. Proceedings of the Annual Interceptor Technology Conference.

[19] Xiao L., Chen A., Cao J., Hou X. (2008). Fast restoration algorithm based on constraint of flight parameters for the turbulence-degraded, i.m.a.g.e.s. Infrared Laser Eng..

[20] Xiao L., Gao L., Ming D., Tian T. (2011). An imaging modeling simulation research of infrared aero-optical effect based on superposition of Gaussian Mixture Model. Proc. SPIE.

[21] Zhang T., Hong H., Sun X., Song Z. (2003). Restoring Turbulence-Degraded Images Based on Estimation of Turbulence Point Spread Function Values. Acta Autom. Sin..

[22] Xiao W., Si-Li G., Fan-Ming L. (2019). Infrared imaging modeling and simulation of aerial target based on BRDF. J. Infrared Millim. Waves.

[23] Alfonsi G. (2009). Reynolds-Averaged Navier–Stokes Equations for Turbulence Modeling. Appl. Mech. Rev..

[24] Moin P., Mahesh K. (1998). Direct numerical simulation: A tool in turbulence research. Annu. Rev. Fluid Mech..

[25] Piomelli U. (1999). Large-eddy simulation: Achievements and challenges. Prog. Aerosp. Sci..

[26] Sundarraj V., Sundarraj K., Kulkarni P.S. (2021). Thermo-Fluid Analysis of Supersonic Flow over Ballistic Shaped Bodies with multiple aero-disk spike configurations. Acta Astronaut..

[27] NOAA (1976). U.S. Standard Atmosphere 1976.

[28] Shih T.-H., Liou W.W., Shabbir A., Yang Z., Zhu J. (1995). A new k-ϵ eddy viscosity model for high reynolds number turbulent flows. Comput. Fluids.

[29] Liou M.-S. (1996). A sequel to ausm: Ausm+. J. Comput. Phys..

[30] Richard H., Raffel M., Rein M., Kompenhans J., Meier G.E.A. (2002). Demonstration of the applicability of a Background Oriented Schlieren (BOS) method. Laser Techniques for Fluid Mechanics.

[31] Shi K., Cheng X., Ma H. (2010). Numerical simulation of aero-optical effects for the flow field around the optical window. J. Infrared Laser Eng..

[32] Pond J.E., Sutton G.W. (2006). Aero-Optic Performance of an Aircraft Forward-Facing Optical Turret. J. Aircr..

[33] Smith R., Truman C., Masson B. Prediction of optical phase degradation using a turbulent transport equation for the variance of index-of-refraction fluctuations. Proceedings of the 28th Aerospace Sciences Meeting.

[34] Yang W., Cai C., Ding M., Zhou C. (2009). Aero-Optic Effects Induced by the Fluctuation Flowfield Surrounding Hypersonic Aircrafts. Acta Photonica Sin..

[35] Gilbert K.G., Otten L.J. (1982). Aero-optical phenomena. J. Prog. Astronaut..

[36] Tabachnick B., Fidell L. (2013). Using Multivariate Statistics.

[37] Abdulredha M., Al Khaddar R., Jordan D., Kot P., Abdulridha A., Hashim K. (2018). Estimating solid waste generation by hospitality industry during major festivals: A quantification model based on multiple regression. Waste Manag..

[38] Hui B., Song Z., Fan H., Zhong P., Hu W., Zhang X., Ling J., Su H., Jin W., Zhang Y. (2019). A dataset for infrared image dim-small aircraft target detection and tracking under ground / air background. Sci. Data Bank.

